# Alcohol and smoking brief interventions by socioeconomic position: a population-based, cross-sectional study in Great Britain

**DOI:** 10.3399/BJGPO.2023.0087

**Published:** 2023-11-29

**Authors:** Vera Helen Buss, Sharon Cox, Graham Moore, Colin Angus, Lion Shahab, Linda Bauld, Jamie Brown

**Affiliations:** 1 Department of Behavioural Science and Health, University College London, London, UK; 2 SPECTRUM Research Consortium, Edinburgh, UK; 3 DECIPHer, School of Social Sciences, Cardiff University, Cardiff, UK; 4 Sheffield Alcohol Research Group, School of Health and Related Research, University of Sheffield, Sheffield, UK; 5 Usher Institute, College of Medicine and Veterinary Medicine, University of Edinburgh, Edinburgh, UK

**Keywords:** general practice, drinking behaviour, smoking, socioeconomic position, cross-sectional studies, substance intervention

## Abstract

**Background:**

Alcohol and smoking brief interventions (BIs) in general practice have been shown to be effective in lowering alcohol and smoking-related harm.

**Aim:**

To assess prevalence of self-reported BI receipt among increasing or higher-risk drinkers and past-year smokers in England, Scotland, and Wales, and associations between intervention receipt and socioeconomic position.

**Design & setting:**

Cross-sectional study using data from a monthly population-based survey in England, Scotland, and Wales.

**Method:**

The study comprised 47 799 participants (15 573 increasing or higher-risk drinkers [alcohol use disorders identification test consumption score ≥5] and 7791 past-year smokers) surveyed via telephone in 2020–2022 (during the COVID-19 pandemic). All data were self-reported. Prevalence of self-reported BI receipt was assessed descriptively; associations between receipt and socioeconomic position were analysed using logistic regression.

**Results:**

Among adults in England, Scotland, and Wales, 32.2% (95% confidence interval [CI] = 31.8 to 32.7) reported increasing or higher-risk drinking and 17.7% (95% CI = 17.3 to 18.1) past-year smoking. Among increasing or higher-risk drinkers, 58.0% (95% CI = 57.1 to 58.9) consulted with a GP in the past year, and of these, 4.1% (95% CI = 3.6 to 4.6) reported receiving BIs. Among past-year smokers, 55.8% (95% CI = 54.5 to 57.1) attended general practice in the past year; of these, 41.0% (95% CI = 39.4 to 42.7) stated receiving BIs. There was a tendency for patients from socioeconomically disadvantaged backgrounds to receive more alcohol (adjusted odds ratio [aOR] 1.38, 95% CI = 1.10 to 1.73) or smoking BIs (aOR 1.11, 95% CI = 0.98 to 1.26), but for the latter the results were statistically non-significant. Results did not differ notably by nation within Great Britain.

**Conclusion:**

BIs in general practice are more common for smoking than for alcohol. A greater proportion of BIs for alcohol were found to be delivered to people who were from socioeconomically disadvantaged backgrounds and who were increasing or higher-risk drinkers.

## How this fits in

It was previously known that delivery rates for alcohol brief interventions (ABIs) in general practice are low, and higher for smoking brief interventions (SBIs). This cross-sectional study found lower delivery rates for both ABIs and SBIs than previous studies. This may be related broadly to changes to general practice during the COVID-19 pandemic. Socioeconomically disadvantaged groups were more likely to have received a brief intervetion (BI).

## Introduction

National Institute for Health and Care Excellence guidelines recommend BIs for alcohol and smoking in primary care (general practice) when appropriate.^1,2^ This is in line with systematic reviews demonstrating the effectiveness of such interventions.^3,4^ For alcohol, GPs can use the alcohol use disorders identification test (AUDIT) to screen their patients for increasing or higher risk of alcohol-related harm.^1^ If they identify patients with scores of ≥8 (≥5 for shorter alcohol use disorders identification test consumption [AUDIT-C] score),^5,6^ guidelines state that GPs should offer BIs on how to reduce alcohol intake.^1^ Similarly, GPs should routinely ask their patients about their smoking status and offer advice on how to quit to current smokers.^2^

Socioeconomically disadvantaged groups experience more alcohol-related harm even though they consume similar or lower levels of alcohol than socioeconomically advantaged groups.^7^ One potential contributor to this divide is worse access to health services,^8^ highlighting the need to monitor BI rates in different socioeconomic groups. In a modelling study,^9^ ABIs (assuming 65% success rate, that is, 3.66 unit-reduction of alcohol per week)^10^ positively impacted health and health inequalities only when they were strongly targeted to the most disadvantaged groups (that is, by increasing intervention rate for residents in more deprived areas). Socioeconomically disadvantaged groups have historically had a higher smoking prevalence and are less likely to be successful in quit attempts compared with people in more advantaged occupations or on higher incomes.^11^ In contrast to ABIs, in a model (assuming 7% of participants in smoking cessation services are abstinent after 1 year, and 4.9% after 2 years),^10^ smoking cessation services had minimal effects on health and health inequalities, even when highly targeted to the most deprived group by increasing the rate of services for them.^9^ The authors explained the difference by pointing out that alcohol-related harm was more strongly associated with deprivation than smoking-related harm.^9^ In contrast, smoking cessation services can have a positive equity effect if more services are offered to socioeconomically disadvantaged groups than to advantaged groups, thereby compensating for lower cessation success in socioeconomically disadvantaged groups.^12^

Previous studies investigating differences in BI receipt by socioeconomic position in England refer to pre-pandemic times.^13,14^ For ABIs, the Scottish Government published data from 2008–2009 on differences according to level of deprivation,^15^ but no equivalent figures for SBIs are available, and there are no data for Wales. Hence, up-to-date information on BI receipt and data comparing rates in England, Scotland, and Wales is scarce, but particularly important given substantial changes to health care following the pandemic.^16,17^ The governments in each nation have taken different approaches to remuneration and setting targets for BIs, with the Scottish Government placing a particular priority on the delivery of ABIs.^10,18^ If the Scottish Government’s efforts resulted in higher rates for ABIs, the English and Welsh governments could learn from the Scottish example and similar efforts could be put into increasing SBI rates. This study aimed to address the following research questions; since the onset of the COVID-19 pandemic:

1) What is the prevalence of self-reported receipt of GP-based ABIs or SBIs among adults with increasing or higher-risk drinking or past-year smokers in England, Scotland, and Wales, and among those who visited their GP in the previous year?

2) To what extent is self-reported receipt of GP-based ABIs or SBIs associated with socioeconomic position (adjusted for demographic and alcohol or smoking-related variables)?

3) Do any associations between self-reported receipt of ABIs or SBIs and socioeconomic position differ between the three nations?

## Method

This study is a population-based, cross-sectional study using data from the Smoking and Alcohol Toolkit Study.^19^ Data for the analyses were collected between October 2020 and October 2022 in England, Scotland, and Wales. This timeframe runs over the three national lockdowns in Great Britain, covering the most acute phases of the COVID-19 pandemic and public health measures taken to address it. The study protocol was published on the Open Science Framework before the analysis.^20^ The manuscript followed the Strengthening the Reporting of Observational Studies in Epidemiology statement.^21^ The data were collected by a market research company and provided in an anonymised form to the research team. Until March 2020, data were collected face to face, but owing to the pandemic, the collection method changed to telephone surveys using the same combination of random location, quota sampling, and weighting technique as before. Studies have demonstrated that the two data collection methods yield comparable results.^22–24^ For the analysis, participants were categorised according to whether they stated that they were at increasing or higher risk of alcohol-related harm, determined by the AUDIT-C score,^5^ or past-year smokers. For increasing or higher-risk drinking, participants had to have an AUDIT-C score of ≥5. For past-year smoking, participants had to smoke cigarettes or other tobacco products or have quit within the past year.

The primary outcome measures were self-reported ABI receipt among all increasing or higher-risk drinkers and those who visited their GP in the past year, and self-reported SBI receipt among all past-year smokers and those who visited their GP in the past year. The reason for including those who stated that they stopped smoking in the past year was that the question about SBI receipt refers to the past year, which means someone might have stopped smoking after receiving an SBI in the past year. First, participants were grouped according to whether they were increasing or higher-risk drinkers or past-year smokers, respectively. Then they were further classified based on whether they had seen their GP and whether they had received BIs. All variables are listed in Table 1 and were self-reported by participants. In sensitivity analyses, England was divided into three regions and social grade into five categories.

**Table 1.** Variables used in analyses, their definitions, and categories or range

Variable	Definition or categories
Increasing or higher-risk drinker	Score ≥5 on AUDIT-C, same cut-off for all^5,6,32^
Past-year smoker	If stated:*‘I smoke cigarettes (including hand-rolled) every day.’* *‘I smoke cigarettes (including hand-rolled), but not every day.’* *‘I do not smoke cigarettes at all, but I do smoke tobacco of some kind (eg, pipe, cigar, or shisha).’* *‘I have stopped smoking completely in the last year*.*‘*
Receipt of alcohol brief intervention	Question:*‘In the last 12 months, has a doctor or other health worker within your GP surgery discussed your drinking?’*If answered:*‘Yes, a doctor or other health worker within my GP surgery offered advice about cutting down on my drinking.’* *‘Yes, a doctor or other health worker within my GP surgery offered help or support within the surgery to help me cut down.’* *‘Yes, a doctor or other health worker within my GP surgery referred me to an alcohol service or advised me to seek specialist help.’*
Receipt of smoking brief intervention	Question:*‘Has your GP spoken to you about smoking in the past year (ie, last 12 months)?’*If answered:*‘Yes, he or she suggested that I go to a specialist stop smoking adviser or group.’* *‘Yes, he or she suggested that I see a nurse in the practice.’* *‘Yes, he or she offered me a prescription for Champix, Zyban, a nicotine patch, nicotine gum or another nicotine product.’* *‘Yes, he or she suggested that I use an e-cigarette.’* *‘Yes, he or she advised me to stop but did not offer anything*.*‘*
GP visit in past year (increasing or higher-risk drinkers)	See *Receipt of alcohol brief intervention* with additional answer option:’*Yes, a doctor or other health worker within my GP surgery asked about my drinking.’*And if replied *‘No’* and answered to follow-up question: *‘I have seen a doctor or health worker within my GP surgery in the last 12 months but did not discuss my drinking.’*
GP visit in past year (past-year smokers)	See *Receipt of smoking brief intervention* with additional answer options:*‘Yes, he or she asked me about my smoking but did not advise me to stop smoking.’* *‘No, I have seen my GP in the last year but he or she has not spoken to me about smoking.’* *‘No, I have not seen my GP in the last year.’*
Gender	Female, male, or non-binary (owing to the small proportion of participants who identified as non-binary, they were excluded from regression analyses)
Age, years	16–24, 25–34, 35–44, 45–54, 55–64, or ≥65
Ethnic group	White or ethnic minority
Nation	England, Scotland, Wales; in sensitivity analysis, England divided into North (North East, North West, and Yorkshire and the Humber), Central (East Midlands, West Midlands, and East of England), and South (London, South East, and South West)
Social grade	Measure of socioeconomic position using the National Readership Survey’s classification^33^ categories: ABC1 (high and intermediate managerial, administrative, or professional; and supervisory, clerical, and junior managerial, administrative, or professional) and C2DE (skilled manual workers; semi and unskilled manual workers; and state pensioners, casual or lowest grade workers, or unemployed with state benefits only); in sensitivity analysis: AB, C1, C2, D, and E
AUDIT-C score	Measure of risk of alcohol-related harm,^5^ range: 5–12
Strength of urge to smoke	Measure of tobacco dependence for past-year smokers,^34^ range: 0–5; if ‘*Not at all*’ [0] selected for *Time with urge to smoke* (*‘How much of the time have you felt the urge to smoke in the past 24 hours?’,* then question: ‘*In general, how strong have the urges to smoke been?’*; answer options: slight [1], moderate [2], strong [3], very strong [4], or extremely strong [5])

AUDIT-C = alcohol use disorders identification test consumption.

The per cent of missing values for each variable are reported in Supplementary Table S1. All values that were noted as ‘Refused’ or ‘Not stated’ by the interviewer were assumed to be missing. A complete-case analysis was conducted. For the first research question, descriptive statistics were used and data were weighted using raking^25^ to match the population of Great Britain (unweighted data in Supplementary Tables S2-S5). The proportion of participants who were eligible for BIs (that is, prevalence of increasing or higher-risk drinking, or past-year smoking), who could have potentially received BIs (that is, eligible people who attended their GP in the past year), and who received a BI (that is, BI receipt among eligible people overall and who attended their GP) in Great Britain and individually in the three nations were assessed. For the other two research questions, regression analyses were performed on unweighted data (analyses using weighted data in Supplementary Table S6). Using logistic regression, unadjusted and adjusted (for age, gender, ethnic group [added after registering the protocol], nation, past-year smoking status and AUDIT-C score for increasing or higher-risk drinkers, and increasing or higher-risk drinking status and strength of urge to smoke for past-year smokers) odds ratios (ORs) were calculated for BI receipt by social grade, for alcohol or smoking separately. For the third research question, an interaction term for social grade and nation was included in the model. A further sensitivity analysis was added that was not specified in the protocol to examine types of interventions offered to increasing or higher-risk drinkers and past-year smokers by their GP in England, Scotland, and Wales (responders were encouraged to select all support options that applied). The analysis was conducted in RStudio (version 2022.07.2, R version 4.2.1).

## Results

Complete data on all relevant variables were available for 47 799 participants. Among these, 32.2% (95% confidence interval [CI] = 31.8 to 32.7) reported increasing or higher-risk drinking and 17.7% (95% CI = 17.3 to 18.1) past-year smoking (see Table 2, unweighted data in Supplementary Table S2). The highest numbers of missing values were recorded for the strength of urge to smoke for past-year smokers (*n* = 415/8825, 4.7%), the AUDIT-C score (*n* = 1803/51 603, 3.5%), and SBIs for past-year smokers (*n* = 226/8825, 2.6%) (see Supplementary Table S1).

**Table 2.** Characteristics of survey responders, self-reported (*n* = 47 799; data weighted)

Characteristic	All, % (95% CI)	England (*n* = 41 106), % (95% CI)	Scotland (*n* = 4167), % (95% CI)	Wales (*n* = 2330), % (95% CI)
Age, years				
18–24	11.7 (11.3 to 12.0)	12.3 (11.9 to 12.7)	8.2 (7.5 to 8.8)	7.6 (6.7 to 8.4)
25–34	17.0 (16.6 to 17.4)	17.4 (16.9 to 17.8)	15.8 (14.9 to 16.7)	12.5 (11.4 to 13.6)
35–44	15.7 (15.3 to 16.1)	15.8 (15.4 to 16.3)	15.4 (14.5 to 16.3)	14.3 (13.1 to 15.5)
45–54	17.0 (16.6 to 17.4)	16.9 (16.5 to 17.3)	18.0 (17.1 to 18.9)	17.0 (15.8 to 18.1)
55–64	15.4 (15.1 to 15.8)	15.0 (14.6 to 15.4)	17.7 (16.9 to 18.6)	18.6 (17.4 to 19.7)
≥65	23.2 (22.8 to 23.6)	22.6 (22.2 to 23.1)	24.9 (24.0 to 25.9)	30.1 (28.7 to 31.5)
Gender				
Female	48.4 (47.9 to 48.9)	48.5 (47.9 to 49.0)	47.9 (46.7 to 49.0)	47.8 (46.3 to 49.4)
Male	51.0 (50.5 to 51.5)	51.0 (50.4 to 51.6)	51.2 (50.1 to 52.4)	51.0 (49.4 to 52.5)
Non-binary	0.6 (0.5 to 0.7)	0.5 (0.5 to 0.6)	0.9 (0.6 to 1.2)	1.2 (0.7 to 1.6)
Ethnic group				
White	86.8 (86.4 to 87.2)	85.5 (85.1 to 85.9)	95.2 (94.7 to 95.7)	95.2 (94.4 to 95.9)
Ethnic minority	13.2 (12.8 to 13.6)	14.5 (14.1 to 14.9)	4.8 (4.3 to 5.3)	4.8 (4.1 to 5.6)
Social grade^a^				
ABC1	56.4 (55.9 to 56.9)	56.8 (56.2 to 57.4)	54.4 (53.2 to 55.5)	52.8 (51.3 to 54.4)
C2DE	43.6 (43.1 to 44.1)	43.2 (42.6 to 43.8)	45.6 (44.5 to 46.8)	47.2 (45.6 to 48.7)
Prevalence of behaviour				
Increasing or higher-risk drinking	32.2 (31.8 to 32.7)	31.9 (31.3 to 32.4)	36.9 (35.8 to 38.0)	30.6 (29.2 to 32.1)
Past-year smoking	17.7 (17.3 to 18.1)	17.8 (17.3 to 18.2)	17.2 (16.3 to 18.0)	17.1 (15.8 to 18.3)

^a^ABC1 = high and intermediate managerial, administrative, or professional; and supervisory, clerical, and junior managerial, administrative, or professional; and C2DE = skilled manual workers; semi and unskilled manual workers; and state pensioners, casual or lowest grade workers, or unemployed with state benefits only.

**Figure 1.**
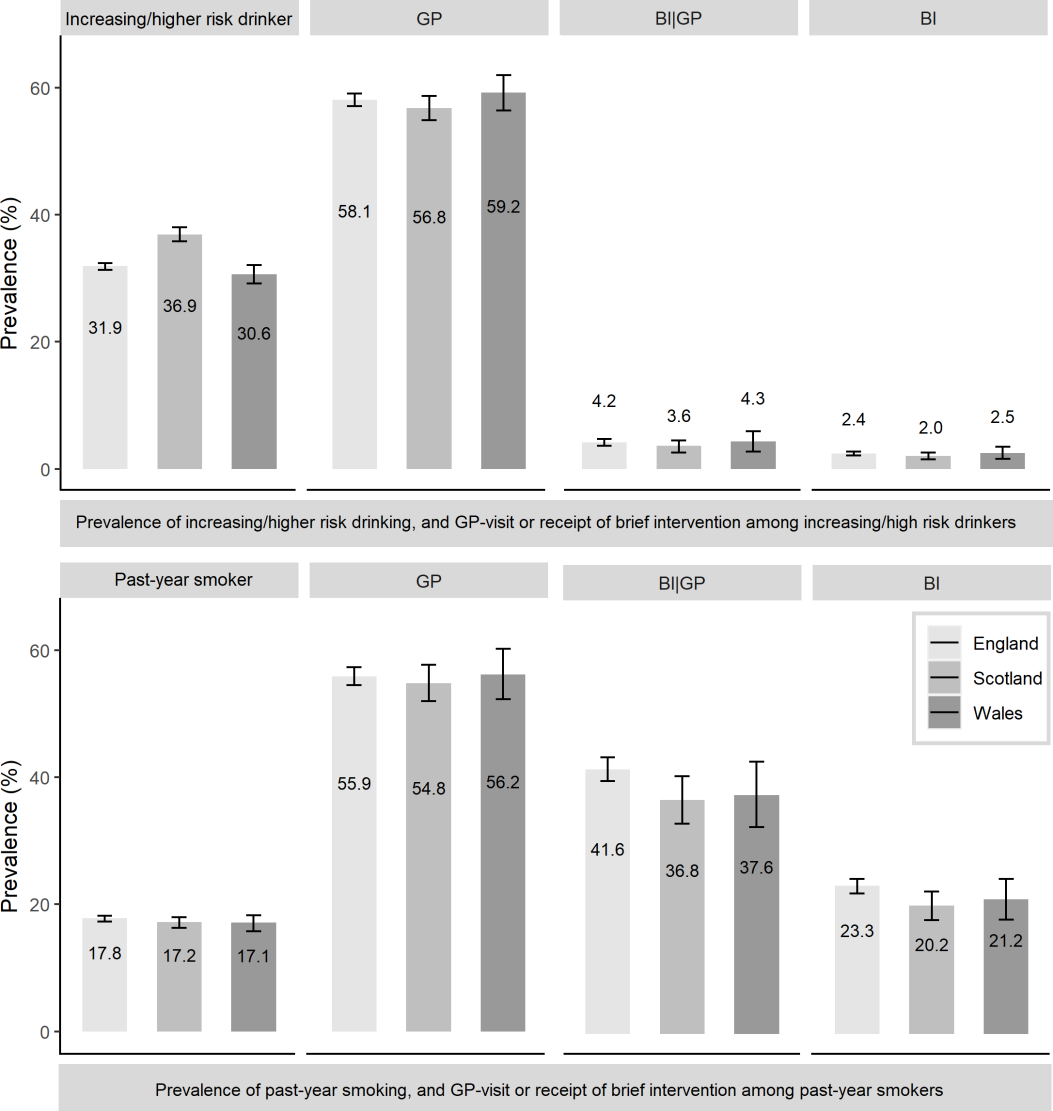
Prevalence of self-reported increasing or higher-risk drinking (top) and past-year smoking (bottom). Among these are those who reported visiting their GP (GP), receiving a BI when visiting their GP (BI|GP), and receiving a BI among all increasing or higher-risk drinkers or past-year smokers (BI); data weighted. BI = brief intervention.

Figure 1 shows the prevalence of self-reported increasing or higher-risk drinking and past-year smoking, GP visits, and BI receipt in all three nations (numbers, percentages, and CIs for weighted and unweighted data can be found in Supplementary Tables S3–S5). In Great Britain overall, 58.0% (95% CI = 57.1 to 58.9) of increasing or higher-risk drinkers consulted with a GP in the past year, and of these, 4.1% (95% CI = 3.6 to 4.6) reported receiving BIs. Among past-year smokers in Great Britain, 55.8% (95% CI = 54.5 to 57.1) attended general practice in the past year; of these, 41.0% (95% CI = 39.4 to 42.7) stated receiving BIs (see Supplementary Table S3). The prevalence of self-reported increasing or higher-risk drinking was the highest in Scotland, while past-year smoking was similar across the nations. In all three nations, fewer increasing or higher-risk drinkers reported receiving BIs from their GP than past-year smokers.

**Figure 2.**
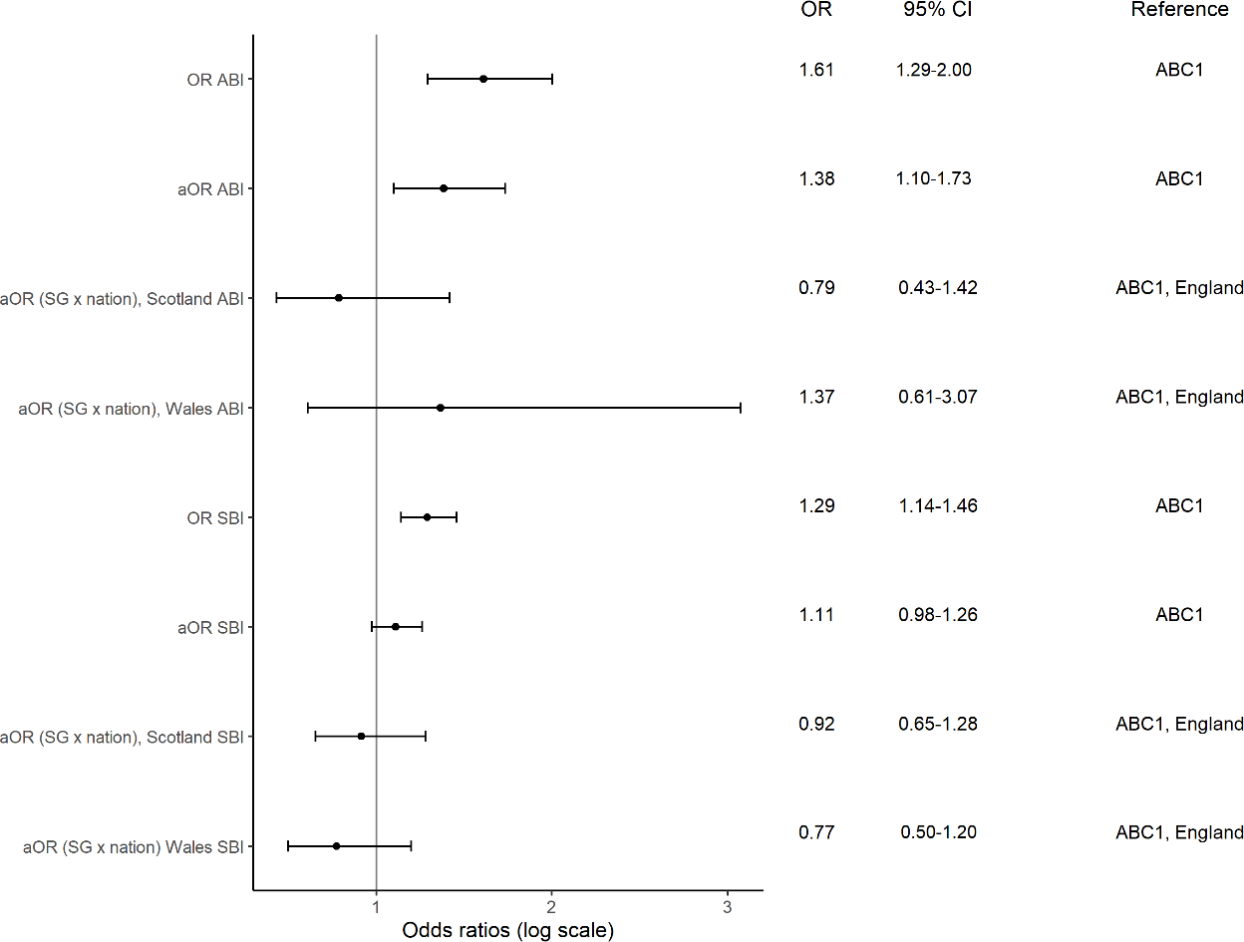
Receipt of BI by socioeconomic position among increasing or higher-risk drinkers (*n* = 9088) and past-year smokers (*n* = 4389) who attended their GP in the past year (data unweighted). ABI adjusted for age, sex, ethnic group, nation, AUDIT-C score, and past-year smoking; SBI adjusted for age, sex, ethnic group, nation, urge to smoke, and increased or higher-risk drinking. All data were self-reported. ABC1 = high and intermediate managerial, administrative, or professional; and supervisory, clerical, and junior managerial, administrative, or professional. ABI = alcohol brief intervention. aOR = adjusted odds ratio. OR = odds ratio. SBI = smoking brief intervention. SG = social grade.

Figure 2 shows the unadjusted and adjusted ORs for receiving ABIs or SBIs by social grade among those who visited their GP in the past year (weighted data in Supplementary Table S6). The unweighted prevalence of receiving ABIs among increasing or higher-risk drinkers attending their GP in the past year was 3.4% (*n* = 216/6410) in the more advantaged social grade group (ABC1) compared with 5.2% (*n* = 140/2678) in the less advantaged (C2DE) (data not shown). For SBIs, the corresponding prevalence rates were 38.1% (*n* = 879/2308) in the more advantaged social grade group and 44.3% (*n* = 921/2081) in the less advantaged. In the unadjusted and adjusted analyses, people with the less advantaged social grade were more likely to have received ABIs or SBIs from their GP. The associations between BI receipt and socioeconomic position did not differ between England, Scotland, and Wales (Figure 1). In the sensitivity analyses (see Supplementary Table S7) using five categories for social grade, the least advantaged (E) had the highest odds of receiving BIs (ABIs: unadjusted OR 2.63, 95% CI = 1.79 to 3.83, aOR 1.91, 95% CI = 1.26 to 2.84; SBIs: unadjusted OR 1.61, 95% CI = 1.30 to 2.00, aOR 1.21, 95% CI = 0.96 to 1.52). The sensitivity analyses (see Supplementary Table S8) with interaction term between social grade and location using five categories did not show any differences.

The sensitivity analysis examining intervention types offered to increasing or higher-risk drinkers and past-year smokers by their GP in Great Britain showed that most increasing or higher-risk drinkers who reported receiving BIs by their GP received advice about cutting down (78.0%, 95% CI = 73.2 to 82.9), and 24.2% (95% CI = 19.0 to 29.4) received a referral to an alcohol service or were advised to seek specialist help. Among past-year smokers who reported having received SBIs by their GP, 42.3% (95% CI = 39.7 to 44.9) recalled receiving advise to stop smoking, and 36.1% (95% CI = 33.6 to 38.7) said they were referred to a specialist stop smoking adviser or group. Responders selected all options that applied. Among those who reported receiving BIs, 17.8% (95% CI = 13.1 to 22.6) of increasing or higher-risk drinkers and 19.9% (95% CI = 17.8 to 22.1) of past-year smokers reported receiving >1 category of support (see Supplementary Table S9).

## Discussion

### Summary

The first objective was to assess the prevalence of self-reported receipt of GP-based ABIs or SBIs among self-reported increasing or higher-risk drinking or past-year smoking since the COVID-19 pandemic. Roughly two in five past-year smokers who visited their GP in the past year reported receiving SBIs. Estimates were similar between England, Scotland, and Wales. The prevalence for self-reported ABI receipt was much lower in all three nations, with about 1 in 25 increasing or higher-risk drinkers who visited their GP in the past year receiving ABIs. The second objective was to examine associations between self-reported BI receipt and socioeconomic position. In unadjusted analyses, those in less advantaged compared with the more advantaged social grades had greater odds of receiving ABIs and SBIs. The associations were reduced by adjusting for other factors and fell slightly below the threshold for statistical significance for smoking, although remained in the same direction. In the sensitivity analysis, including five social grade categories, the least advantaged, representing those on low incomes and those with recourse to state benefits, had the highest odds of receiving BIs compared with the most advantaged. Regarding the third objective, no differences were found between the three nations and the association between self-reported GP-based BI receipt and socioeconomic position. The sensitivity analysis, which examined the type of intervention received, showed 24.2% of increasing or higher-risk drinkers and 36.1% of past-year smokers, who each reported having received BIs by their GP, stated that they were referred to a specialist service or advised to seek specialist help.

### Strengths and limitations

To the authors’ knowledge, this is the first study comparing ABI and SBI data from England, Scotland, and Wales. A further strength is the large representative sample with only few missing values. Among the limitations are potential self-reporting and recall biases, small sample sizes for subanalyses (for example, for BI receipt in Scotland and Wales), and only social grade as a measure of socioeconomic position. Further, the study is based on cross-sectional observational data and potential covariates may not have been measured. For potential SBI receipt, participants were included who reported having stopped smoking in the past year (weighted number of past-year smokers *n* = 8414 versus current smokers *n* = 7351). It is possible that these may have visited their GP after having stopped smoking, which would have made them ineligible to receive SBIs at the time of the GP consultation. By implication, the results are conservative, possibly underestimating SBI receipt. This limitation is owing to the cross-sectional study design.

### Comparison with existing literature

The results are comparable with previous research showing that smokers in England are substantially more likely to report receiving GP-based BIs than increasing or higher-risk drinkers.^13,26^ This study has shown that these findings are also applicable to Scotland and Wales. However, the proportion of patients reporting receiving BIs has declined. Data from March–November 2014 showed 50.4% (95% CI = 48.0 to 52.8) of past-year smokers and 6.5% (95% CI = 5.1 to 7.9) of increasing or higher-risk drinkers visiting their GP in the past year (62.1% and 58.9%, respectively, visited GPs) reported receiving BIs in England.^26^ From March 2014–July 2016, 48.3% (95% CI = 47.1 to 49.5) of past-year smokers and 6.1% (95% CI = 5.4 to 6.5) of increasing or higher-risk drinkers in England attending general practice (54.9% and 64.8%, respectively, visited GPs) recalled receiving BIs.^13^ An analysis that only included past-year smokers in England found that 47.2% (95% CI = 46.1 to 48.3) stated their GP had offered brief advice, based on data collected between September 2016 and October 2019.^14^ The present study, which comprised a two-year period from October 2020, found a prevalence of 41.6% (95% CI = 39.8 to 43.5) for past-year smokers and 4.2% (95% CI = 3.6 to 4.7) for increasing or higher-risk drinkers in England with GP visits (55.8% and 58.0%, respectively, visited GPs), with similar values for the other two nations. A possible explanation for the decline in the delivery of BIs in primary care is the COVID-19 pandemic that began before this study started, while all other studies pre-dated the pandemic. It is known that the pandemic disrupted routine delivery of primary care services, including preventive care and health promotion services.^27^ The prevalence of increasing or higher-risk drinking increased compared with pre-pandemic levels (for England, 32% in this study and 13% previously).^13^ The rapid increase during the pandemic could also have played a role as GPs may not have picked it up so quickly, resulting in lower advice rates. Regarding socioeconomic differences, similar to this analysis, a previous study found a socioeconomic gradient in BI receipt, which was more pronounced for alcohol than for smoking.^13^ A US study found women had significantly lower odds of receiving ABIs in primary care than men.^28^ The present study did not assess sex differences, but it could be the focus of future work.

### Implications for research and practice

Possible explanations for the low rates of ABIs are that increasing or higher-risk drinkers may not recognise their drinking as a problem and/or the stigma associated with alcohol problems, which has been reported to be a barrier to offering and accepting support.^29,30^ Surprisingly, the proportion of increasing or higher-risk drinkers who reported receiving GP-based ABIs was not higher in Scotland compared with the other nations considering the efforts the Scottish Government and public health agencies have made in recent years to reduce alcohol-related harm.^18^ The Scottish Government has reported figures above target for the delivery of ABIs since 2011–2012.^18^ In the latest published figures from 2019–2020, 52.7% of the 75 616 ABIs in Scotland were delivered in primary care.^31^ The discrepancy between the official statistics and this study’s results could indicate different understandings of what constitutes BIs between GPs and patients. However, unsuccessful interventions can be assumed if the people surveyed stated that they had not received one, even if — from the GP’s view — an intervention was delivered. It is also possible that participants were more truthful in reporting their drinking behaviour to the interviewer than to their GP, which would mean that GPs may not be aware of their patients’ problematic drinking. The study results have suggested that while SBIs neither widen nor reduce the inequality gap, ABIs may help narrow the gap as people from the least advantaged social grade were significantly more likely to have received BIs than those from the most advantaged.

## Data

Data are available on request. The command syntax for the statistical analyses is available on GitHub (https://github.com/UCL-Tobacco-and-Alcohol-Research-Group).
